# STED Super-Resolution Microscopy of Clinical Paraffin-Embedded Human Rectal Cancer Tissue

**DOI:** 10.1371/journal.pone.0101563

**Published:** 2014-07-15

**Authors:** Peter Ilgen, Stefan Stoldt, Lena-Christin Conradi, Christian Andreas Wurm, Josef Rüschoff, B. Michael Ghadimi, Torsten Liersch, Stefan Jakobs

**Affiliations:** 1 Department of NanoBiophotonics, Max Planck Institute for Biophysical Chemistry, Göttingen, Germany; 2 Department of General, Visceral and Pediatric Surgery, University Medical Center of Göttingen, Göttingen, Germany; 3 Institute of Pathology Nordhessen, Kassel, Germany, and Department of Pathology, University Medical Center of Göttingen, Göttingen, Germany; 4 Department of Neurology, University Medical Center of Göttingen, Göttingen, Germany; 5 Center Nanoscale Microscopy and Molecular Physiology of the Brain (CNMPB), Göttingen, Germany; The Chinese University of Hong Kong, Hong Kong

## Abstract

Formalin fixed and paraffin-embedded human tissue resected during cancer surgery is indispensable for diagnostic and therapeutic purposes and represents a vast and largely unexploited resource for research. Optical microscopy of such specimen is curtailed by the diffraction-limited resolution of conventional optical microscopy. To overcome this limitation, we used STED super-resolution microscopy enabling optical resolution well below the diffraction barrier. We visualized nanoscale protein distributions in sections of well-annotated paraffin-embedded human rectal cancer tissue stored in a clinical repository. Using antisera against several mitochondrial proteins, STED microscopy revealed distinct sub-mitochondrial protein distributions, suggesting a high level of structural preservation. Analysis of human tissues stored for up to 17 years demonstrated that these samples were still amenable for super-resolution microscopy. STED microscopy of sections of HER2 positive rectal adenocarcinoma revealed details in the surface and intracellular HER2 distribution that were blurred in the corresponding conventional images, demonstrating the potential of super-resolution microscopy to explore the thus far largely untapped nanoscale regime in tissues stored in biorepositories.

## Introduction

The number of human tissue specimen stored in (clinical) biorepositories was estimated at more than 300 million already 15 years ago in the US alone [1], [2]. Many of these specimens have been formalin fixed and paraffin embedded, a standard clinical preservation method that is in use for more than a century. In many clinical settings, tissue taken from cancer patients during oncological surgery is stored in blocks of paraffin for diagnosis, decision on postoperative treatment strategies and follow-up studies, representing a vast and valuable resource to study the pathology and functional basis of many malignancies.

For analyses, the stored and annotated paraffin-embedded tissue may be sectioned, dewaxed, stained and then imaged by conventional light microscopy. In classical (fluorescence) microscopy the attainable resolution is limited by diffraction to about 250 nm, restricting the extractable information from a specimen. Over the last decade, several super-resolution microscopy techniques (nanoscopy) have been developed that allow to fundamentally overcome the diffraction limit, enabling far-field microscopy with a substantially improved resolution [3]–[5].

Of these techniques, stimulated emission depletion (STED) microscopy stands out as an approach that may be used in conjunction with immunolabeled samples and that does not require computational efforts to generate the final image [6], [7]. In STED microscopy, a light pattern is used to inhibit fluorescence at well-defined sample coordinates such that adjacent features emit sequentially. The inhibition of fluorescence is accomplished by stimulated emission of excited fluorophores to the ground state. In a typical STED microscopy implementation the fluorophores located at the outer rim of a scanning focal spot of excitation light are transiently switched off by de-excitation through stimulated emission with a doughnut-shaped STED-beam featuring a central zero. As a consequence, only fluorophores within an effective focus with a diameter of 
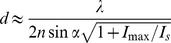
 are able to fluoresce. Here, *λ* is the wavelength, *I_s_* is a characteristic of the fluorophore, and *I_max_* denotes the intensity of the peak enclosing the zero. For *I_max_*/*I_s_*>>1, the effective focus becomes much smaller than the diffraction limit. As a consequence, unlike in conventional lens-based optical microscopes, the resolution is no longer limited by the wavelength.

STED microscopy hence lends itself as a powerful tool to open up the thus far largely untapped nanoscale regime in archived human tissue material. Previously, various super-resolution techniques have been utilized to image protein localizations in chemically fixed vibratome sections or cryostat sections of mouse or rat tissues [8]–[10], as well as in sections of plastic embedded tissues from rodents expressing fluorescent proteins as tags [11], [12]. STED microscopy has also been used to image living tissue [13]–[15]. None of these approaches could immediately be transferred into established clinical procedures which require standardized procedures and the possibility for long term sample storage.

To investigate if STED microscopy can be used to visualize protein distributions in paraffin-embedded tissue, we used archived rectal cancer tissue that had been resected during standardized total mesorectal excision (TME-surgery) and was stored at room temperature for up to 17 years in a clinical pathology repository. This tissue was chosen for this study because with a continuously increasing incidence, colorectal cancer has become one of the three most frequent cancers in the western world [16]. One third of the colorectal cancers are rectal cancers and despite of advances in diagnostics and multimodality treatment using pre-operative (neoadjuvant) chemoradiotherapy followed by extended oncological TME-surgery, the long-term prognosis of patients with locally advanced rectal cancer is limited by the occurrence of distant metastases in nearly 30% of patients [17]–[19].

Deregulation of signaling through the human epidermal growth factor receptor (HER; also known as ERBB) family of proteins has an intricate role in the pathogenesis of numerous human cancers [20]. Recently, it was demonstrated that HER2 is overexpressed in ∼30% of primary locally advanced rectal adenocarcinoma, clinically staged as Union for International Cancer Control (UICC) stages II and III [21]. Little is known on the subcellular localization of HER2 in tumor tissues.

In this manuscript we investigated the feasibility of visualizing the nanoscale distribution of HER2 and other proteins using STED super-resolution microscopy in archived rectal cancer tissue. We demonstrate that even tissues stored for more than a decade in a clinical repository are amenable for STED super-resolution imaging.

## Results

### Fluorescence microscopy of thin sections

**Figure 1:**
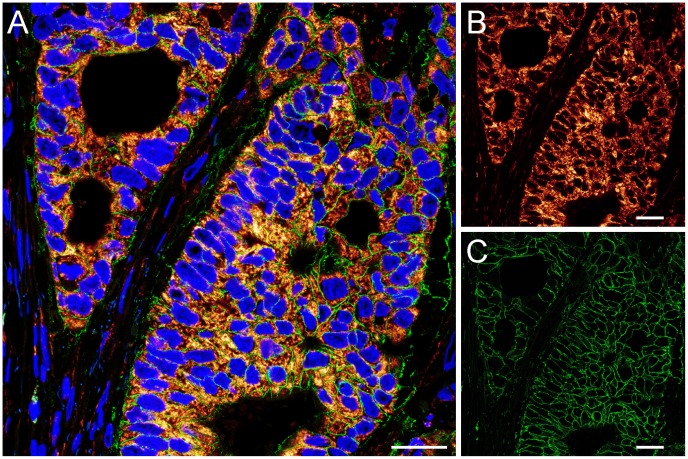
Immunofluorescence labeling of HER2 positive paraffin-embedded rectal cancer tissue. Confocal overview image of a region of a HER2 positive rectal cancer tissue section labeled with DAPI (blue) to highlight the nuclei and decorated with antisera against Tom20 (fire) and HER2 (green). (A) overlay, (B) Tom20, and (C) HER2. Scale bars: 25 µm.

HER2 positive rectal carcinoma tissue, archived after formalin fixation and paraffin-embedment, was sectioned into 2 µm thick slices. The tissue sections were dewaxed and heat treated for 45 min for antigen retrieval [22]. After this, the sections were stained with DAPI to highlight the nuclei and decorated with antisera against the integral membrane protein HER2 and against the mitochondrial outer membrane protein Tom20, an essential protein expressed in all human cells. Confocal microscopy demonstrated the strong expression of HER2 in the tumor tissue, whereas the surrounding normal stroma cells did not express a relevant amount of HER2 (Figure 1). Mitochondrial mass was higher in the malignant cells and as a consequence Tom20 was more abundant, although, as expected, Tom20 signals were also detectable in the normal stroma cells. Also the DAPI signal was recognizable both in cancer cells and normal tissue. We conclude that multi-color immunofluorescence labeling and confocal microscopy is feasible on archived routinely processed clinical paraffin-embedded tissue.

### Structural preservation on the nanoscale

The optical resolution of conventional confocal microscopy is limited by diffraction to ∼200 nm in the axial plane, often concealing valuable information. Paraffin-embedded tissues allegedly exhibit pronounced autofluorescence [23] and, although the attainable structural preservation of paraffin-embedded tissue is sufficient for conventional light microscopy (Figure 1; [24]), archived tissues may be unsuitable to meet the high demands of diffraction unlimited super-resolution microscopy. To investigate if routinely processed stored paraffin-embedded clinical human tissue is suitable for STED microscopy, we decided to analyze sub-mitochondrial protein distributions in mitochondria. These organelles are challenging cellular structures for super-resolution microscopy due to their small size, their complex inner organellar architecture and the very dense packaging of proteins in the mitochondrial membranes [25]. To this end, we utilized paraffin-embedded rectal cancer tissue that was stored for ∼1 year at room temperature in a clinical pathology archive. Tissue sections containing a longitudinal section of the inner circular muscle layer of the rectum (*Muscularis externa*) were decorated with antisera against four different mitochondrial proteins: Tom20, a peripheral receptor of the TOM translocase in the mitochondrial outer membrane; Mic60 (mitofilin), a component of the MINOS complex localized preferentially at the cristae junctions in the inner membrane; aconitase, a matrix enzyme of the tricarboxylic acid cycle catalyzing the isomerization of citrate to isocitrate; and cyclophilin D, a peptidylprolyl isomerase localized in the mitochondrial matrix.

**Figure 2:**
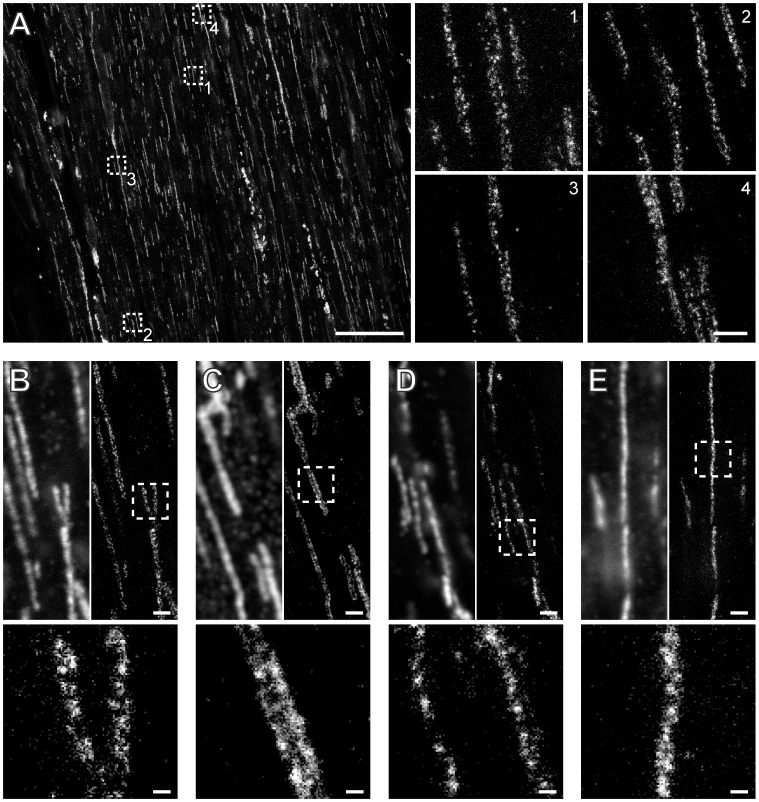
STED super-resolution microscopy of mitochondria in the rectal *Muscularis externa* demonstrates high structural preservation of the stored paraffin-embedded tissue. STED recordings were performed on 2 µm thick dewaxed sections cut along the longitudinal axis of the rectum. (A) Left: STED overview image of a region of the inner circular layer of the rectal *Muscularis externa* decorated with an antiserum against Tom20. Right: Magnifications of the areas in the indicated dashed squares showing the distribution of TOM clusters within the mitochondria. (B–E) STED images of tissue sections decorated with antisera against Tom20 (B), Mic60 (mitofilin) (C), aconitase (D), and cyclophilin D (E). In each panel the confocal (top, left) and the corresponding STED image (top, right) is displayed. Bottom: Magnification of the STED image as indicated by a dashed square. Note the different distributions of the four proteins within the mitochondria. Scale bars: 20 µm (A, left); 1 µm (A, right) and (B–E, top); 200 nm (B–E, bottom).

The mitochondria in the *Muscularis externa* form large elongated tubules. When the tissue blocks were cut along the longitudinal axis of the resected rectal specimen, the majority of the organelles was stretched out within the section plane (Figure 2A). Across a large STED image of size 120 µm×100 µm, Tom20 showed a distinct punctate localization within the mitochondria (Figure 2A), which is in full agreement with previous studies using various forms of super-resolution microscopy in cultured mammalian cells [26], [27]. The attained resolution, as determined on background-clusters, was consistently ∼40 nm throughout the recorded tissue sections (Figure S1). Whereas in the corresponding confocal recordings the distributions of Tom20, Mic60, aconitase and cyclophilin D were practically indistinguishable, the higher resolution provided by the STED microscope demonstrated differences in sub-mitochondrial distributions of the respective proteins (Figure 2B-E). Tom20, aconitase and cyclophilin D were generally found in evenly distributed clusters, albeit in different amounts. Sections decorated with an antiserum against cyclophilin D exhibited the densest clustering (Figure 2E), whereas an antiserum against aconitase resulted in the labeling of sparse clusters (Figure 2D). As shown previously for Mic60 in cultured cells [28], this protein appears to have also a more ordered distribution in human tissue (Figure 2C). The overall sub-mitochondrial distribution of the respective proteins in the tissue was comparable to the localization observed in cultured human cells (Figure S2).

Taken together, these data suggest a sufficient structural preservation of routine clinical paraffin-embedded tissue qualifying it for use in STED super-resolution microscopy.

### STED super-resolution microscopy of HER2 labeled rectal cancer tissue

**Figure 3:**
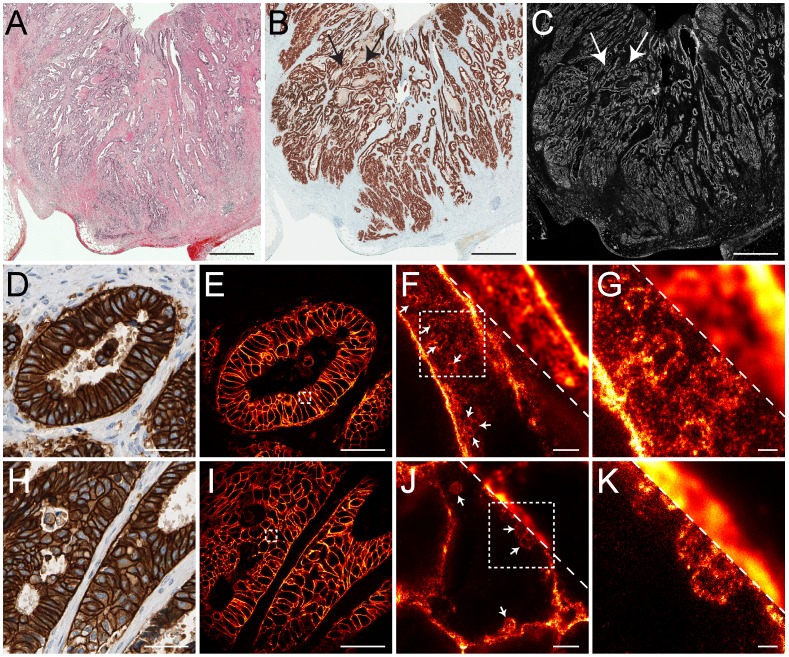
Comparison of (A) Hematoxylin-Eosin-, (B) immunohistochemistry-, and (C) immunofluorescence-labeling on three consecutive 2 µm thick tissue sections of a HER2 positive paraffin-embedded human rectal cancer. Note that the three consecutive sections cover the same region in the tissue. The images were taken with diffraction-limited widefield (A,B) or confocal (C) microscopy. (D,H) and (E,I): corresponding magnifications out of (B) and (C), respectively, at sites indicated by the arrows. (F,G,J,K): comparison of diffraction-limited confocal microscopy with STED super-resolution microscopy on sectioned stored tumor tissue. Shown are magnifications of the areas indicated by the dashed squares. Top-right corners: confocal imaging. Left-bottom area: STED imaging. The arrows point to HER2-positive vesicle-like structures that are resolved in the STED images. Scale bars: 1 mm (A–C), 50 µm (D,E,H,I), 2 µm (F,J), and 500 nm (G,K).

In order to evaluate the benefit of STED microscopy over conventional microscopy, we visualized the distribution of HER2 in sections of rectal cancer tissue that was scored as HER2-positive based on immunochemistry and in situ hybridization [21], [29]. Three consecutive 2 µm-thick sections of the paraffin-embedded tissue were cut and treated alike for dewaxing and antigen retrieval. The three sections cover the same region, allowing to directly compare the properties of different staining procedures. The first section was stained by a standard clinical Hematoxylin-Eosin (HE) procedure. As a result, the nuclei were stained in blue, whereas the cytoplasm and the extracellular matrix appeared in various shades of pink (Figure 3A). The other two sections were decorated with an antiserum against HER2 but with different secondary antibodies. For standard immunohistochemistry, we used a secondary antibody conjugated with peroxidase, catalyzing the oxidation of 3, 3′-diaminobenzidine, resulting in a brown precipitate (Figure 3B). For fluorescence microscopy, secondary antibodies labeled with the red fluorescent dye KK114 [30] were used (Figure 3C).

The HE staining provides an overview of the tissue, whereas the immunohistochemistry and immunofluorescence images show strong HER2 expression in the tumor cells, but not in the surrounding non-malignant stroma tissue. Next, we compared the immunohistochemistry staining with the immunofluorescence labeling as recorded by confocal and STED microscopy (Figure 3B-K). To this end, we selected corresponding regions in the two differently labeled tissue sections. Whereas the confocal images of the immunofluorescence labeling provide a higher contrast and the membranes are more sharply delineated as in the images of the immunohistochemistry staining, the overall information content appears comparable (Figure 3D,E,H,I). Presumably, the immunofluorescence image appears sharper, because in case of immunohistochemistry the achievable resolution is ultimately limited by the diffusion of the brown precipitate. Both approaches demonstrate that HER2 is predominantly found in the plasma membrane of rectal cancer cells. STED microscopy enables the visualization of additional details, which are obscured in the diffraction-limited confocal images (Figure 3F,G,J,K; Figure S3). The STED image clearly shows ∼450 nm sized HER2 positive vesicle-like structures, which may be due to HER2 internalization. The STED images even suggest the existence of individual HER2 protein clusters, which are fully concealed in the diffraction-limited conventional images.

We conclude that the sub-cellular distribution of HER2 can be visualized in sections of archived clinical paraffin-embedded rectal cancer tissues. STED microscopy reveals structural details, including HER2 positive vesicle-like structures in the stored material that are blurred when using state-of-the-art conventional light microcopy.

### STED super-resolution microscopy on archived clinical material after long-term storage

**Figure 4:**
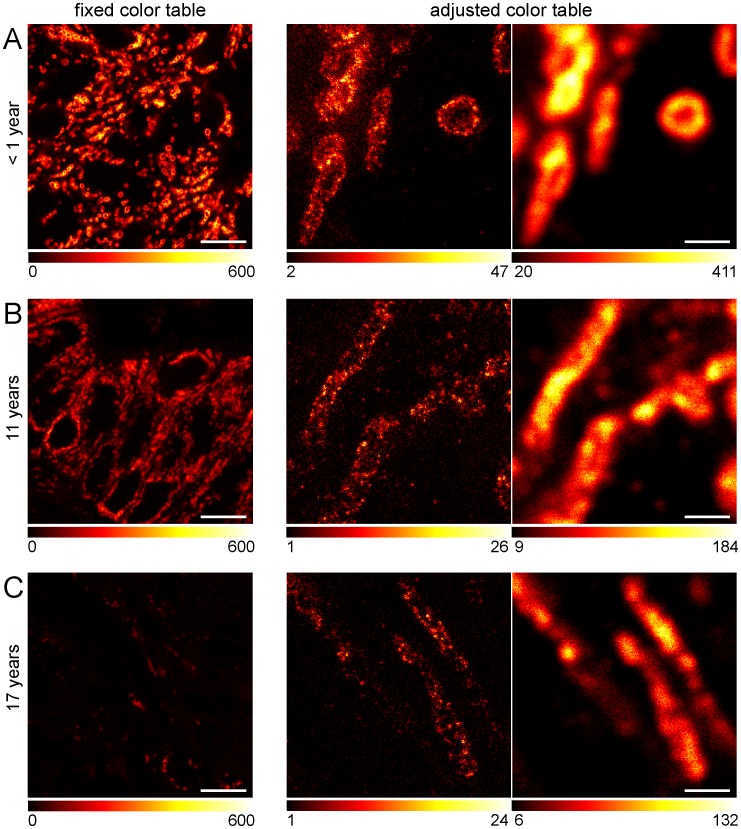
STED super-resolution microscopy of archived human tissue samples stored for up to 17 years in a clinical repository. Representative images of tumor tissues stored at room temperature for less than 1 year (A), 11 years (B) or 17 years (C), were sectioned, dewaxed, decorated with an antiserum against Tom20 and imaged. Left: Representative confocal images. The same color table was used for the three images in order to visualize the relative staining efficiencies. Middle/Right: Comparison of STED (middle) and confocal (right) microscopy of tissue sections of different age. Here, the color tables were adjusted to the signal intensities obtained. Scale bars: 10 µm (left) and 1 µm (middle, right).

The potential of super-resolution microscopy on paraffin-embedded tissue can only be explored widely if the method could be used on standard clinical samples as they are stored in numerous clinical archives around the world. To determine the influence of prolonged storage on the usability of the archived tissue for STED microscopy, we used paraffin-embedded, rectal cancer tissues that were stored at room temperature in a routine clinical pathology archive for up to 17 years. The paraffin-embedded tissues were pretreated as before and labeled with antibodies against the mitochondrial protein Tom20 (Figure 4). We found that even 17 years old tissue could be used for immunofluorescence labeling, although with increasing storing time the brightness of the labeled sections was reduced, presumably reflecting a decrease of accessible epitopes in the aged samples. Remarkably, even in the oldest tissue sample, individual Tom20 clusters could be resolved by STED microscopy, which were not discernible in the respective corresponding diffraction-limited confocal images, evidencing that even decades old paraffin-embedded human tissues are amenable for STED super-resolution microscopy.

## Discussion

Cancer tissue resected during oncological surgery is very important for diagnostics. For routine analysis, the expression levels of specific key proteins are generally determined on the tumor or cellular level. Proteins, however, execute their functions locally, and hence the nanoscale distribution of key proteins might contain valuable signatures, which have thus far not been considered for clinical diagnosis. In this study we show that STED super-resolution microscopy is suited to reveal nanoscale protein distributions in tissues stored for decades in biorepositories, thus opening up the nanoscale in these specimens.

There is an enormous and fast growing amount of tissue samples stored in various biobanks. Biospecimen quality is considered as a critical issue [2], [31] and the quality of the specimen will also be critical for the data that can be extracted by super-resolution microscopy. Nonetheless, due to practical constrains, the structural preservation of specimen resected during routine surgery is unlikely to have the quality level that is achievable under strictly controlled laboratory conditions. There are further potential, although presumably surmountable, challenges including the quality and availability of suitable antibodies and the challenge to combine imaging data with, for example, metabolic signatures of the samples. We envision that future developments in the super-resolution field including the parallelization of image acquisition [32], [33] as well as automated image analysis will enable large scale analysis of biobanked tissues using STED and other super-resolution techniques.

## Materials and Methods

### Ethics Statement

The study was approved by the medical ethics committee of the University of Göttingen (June 28, 2006; #9/8/08). The origin of the specific samples used in this study has been described in a previous publication [21].

### Tissue fixation and embedding

Freshly resected tissue was fixed in 4.5% buffered formalin (Th. Geyer, Renningen, Germany) at room temperature for 12 to 24 h. The fixed tissue was embedded automatically in paraffin (Süsse Labortechnik, Gudensberg, Germany) using a tissue processor (Leica ASP 300; Leica Biosystems, Wetzlar, Germany). The paraffin blocks were stored in the dark at room temperature.

### Preparation of tissue slides

Paraffin blocks were cooled down to −10°C on a cooling plate (PFM Medical AG, Cologne, Germany). The cooled paraffin blocks were cut into 2 µm thick tissue sections on a microtome (HM 430, MICROM International, Walldorf, Germany) and transferred with a brush into water at room temperature before being mounted onto glass slides. To stretch the sectioned tissue samples, the glass slides were placed on a heating table (MEDAX, Kiel, Germany) at 37°C and dried in a heating incubator at 37°C overnight.

### Dewaxing

The dried slides were deparaffinized with Xylol (2×8 min) followed by a series of descending alcohol concentrations (2×2 min 100% EtOH, 2×2 min 96% EtOH, 1×2 min 75% EtOH) and finally rinsed 3 times in water.

### Automated immunohistochemistry

Standardized immunohistochemical staining was performed in a Ventana BenchMark XT Immunostainer (Ventana, Ventana Medical Systems, Mannheim, Germany). Using the immunostainer, the heat epitope retrieval was automatically performed on tissue sections in CC1 (Cell Conditioning 1 solution, Ventana Medical Systems, Mannheim, Germany) buffer for 60 min at 100°C. Subsequently, the tissue sections were automatically incubated with the PATHWAY anti-HER-2/neu (4B5) rabbit monoclonal antibody at 37°C for 32 min. Then the sections were decorated with secondary antibodies coupled to horseradish peroxidase and stained with 3,3′-diaminobenzidine (ultraView Universal DAB Detection Kit; Ventana Medical Systems, Mannheim, Germany). As counterstain, the Hematoxylin II counterstain reagent (Ventana Medical Systems, Mannheim, Germany) was used to stain nuclei. Finally, the object slides were manually dehydrated in a series of ascending alcoholic concentrations (2×1 min 75% EtOH, 2×1 min 96% EtOH, 2×1 min 100% EtOH) with a final 2 min incubation in Xylol. The slides were mounted in Vitro-Clud (Langenbrinck, Labor- und Medizintechnik, Emmending, Germany). This method was used for Figure 3B,D,H.

### Manual Hematoxylin and eosin (HE) staining

HE staining was performed on sections of deparaffinized tissue. The tissue slides were rinsed in water and then submerged in Hemalum solution (Carl Roth GmbH + Co. KG, Karlsruhe, Germany) for 10 min. To allow the development of the blue color of the stained nuclei, the tissue slides were rinsed in tab water for 10 min. Subsequently, the samples were counterstained for 30 seconds in an Eosin solution (1% Eosin in 37.5% EtOH, Merck Millipore), rinsed in pure H_2_O and dehydrated in a series of ascending alcoholic concentrations (2×1 min 75% EtOH, 2×1 min 96% EtOH, 2×1 min 100% EtOH) with a final 2 min incubation in Xylol. The slides were mounted in Vitro-Clud (Langenbrinck, Labor- und Medizintechnik, Emmending, Germany). This method was used for Figure 3A.

### Manual antigen retrieval

Sections were transferred into a slide chamber, submerged in CC1 solution (Cell Conditioning 1 solution, Ventana Medical Systems, Mannheim, Germany) and then heated for 45–60 min at 100°C in a steamer (Multi Gourmet plus, Braun, Kronberg, Germany). After cooling for 20 min, slides were rinsed in water.

### Manual immunolabeling

Sections were blocked with 5% or 10% (wt/vol) BSA in PBS (137 mM NaCl, 3 mM KCl, 8 mM Na_2_HPO_4_, 1.5 mM KH_2_PO_4_, pH 7.4) for 5 min and incubated for 1 h with the primary rabbit monoclonal antibody PATHWAY anti-HER-2/neu (4B5) (Ventana Medical Systems, Mannheim, Germany; Catalog Number:790–2991) (dilution: 1: 50), polyclonal rabbit antibodies against Tom20 (Santa Cruz Biotechnology, Santa Cruz, USA; sc-11415) (dilution: 1∶200), polyclonal rabbit antibodies against Mic60/mitofilin (Abcam, Cambridge, United Kingdom; ab48139) (dilution: 1∶200), polyclonal rabbit antibodies directed against aconitase (Sigma Aldrich, St. Louis, MO, USA; HPA001097) (dilution: 1∶400), or monoclonal antibodies against cyclophilin D (also named Cyclophilin 3 or Cyclophilin F; Abcam, Cambridge, UK; ab110324) (dilution: 1∶400), respectively, at RT. The primary antibodies were detected with secondary antibodies (sheep anti-mouse or goat anti-rabbit; Jackson ImmunoResearch Laboratories) custom labeled with the red emitting dye KK114 [30] (dilution: 1∶200) or a secondary antibody (sheep anti-mouse; Dianova, Hamburg, Germany) custom labeled with the yellow emitting dye Atto532 (ATTO-TEC, Siegen, Germany) (dilution: 1∶100). After immunolabeling, the tissue samples were mounted in Mowiol supplemented with 0.1% (wt/vol) DABCO (1,4-diazabicyclo [2.2.2]octane; Sigma Aldrich, St. Louis, MO, USA) and 2.5 µg/mL DAPI (4′,6-diamidino-2-phenylindole; Sigma-Aldrich).

### Microscopy

All images shown represent representative images. Sections from more than 10 different paraffin blocks were analyzed, persistently providing similar results. The immunohistochemistry-stained and HE-stained tissue sections were imaged with an Axio Imager 2 equipped with an Axio-Cam MRc (Zeiss, Jena, Germany). For confocal microscopy, a Leica TCS SP5 microscope was used (Leica Microsystems, Wetzlar, Germany). For STED and the corresponding confocal microscopy, a custom built STED microscope was used [26], [34]. A resolution of ∼250 nm in the confocal images and ∼40 nm in the STED images was achieved. The resolution was determined by measuring the full width at half maximum (FWHM) in the x and the y axis on more than 100 background clusters, which presumably stem from precipitated secondary antibodies. Imaging was performed essentially as described previously [26], [34]. Except for contrast stretching no further image processing was applied.

## Supporting Information

Figure S1 **Measured diameter of background clusters taken from** **Figure 2** **.** (A) The full width at half maximum (fwhm) across the x and the y axes of more than 100 individual clusters was determined. Note that also background clusters that were physically larger than the resolution of the microscope were analyzed. Therefore the determined average fwhm may be worse than the actual resolution of the microscope used. (B) Representative background clusters. Scale bar: 100 nm.
(TIF)

Figure S2 S**ub-mitochondrial protein distributions in cultured human cells recorded with a STED (left) and confocal (right) microscope.** Detail of mitochondria decorated with antisera against Tom20 (A), aconitase (B), Mic60/mitofilin (C), or cyclophilin D (D). Human primary fibroblasts (A,C) or U2OS (human bone osteosarcoma epithelial cells) (B,D). Scale bars: 500 nm.
(TIF)

Figure S3 **Line profile through a region taken from** **Figure 3F** **.** The STED super-resolution image (A) reveals HER2 positive vesicle-like structures that are blurred in the corresponding confocal image (B). (C) Normalized fluorescence signal intensity profiles along the indicated lines in the STED (red) and the confocal (black) images. The scatter plot shows the fluorescence signals averaged over 3 adjacent intensity profiles. The solid lines represent the corresponding fits using a Lorentz function. Scale bar: 500 nm.
(TIF)
