# Supplementary figures and images for: STED Super-Resolution Microscopy of Clinical Paraffin-Embedded Human Rectal Cancer Tissue
Source: PLoS One. 2014 Jul 15;9(7):e101563. doi: 10.1371/journal.pone.0101563 (PMC4099123; doi:10.1371/journal.pone.0101563)

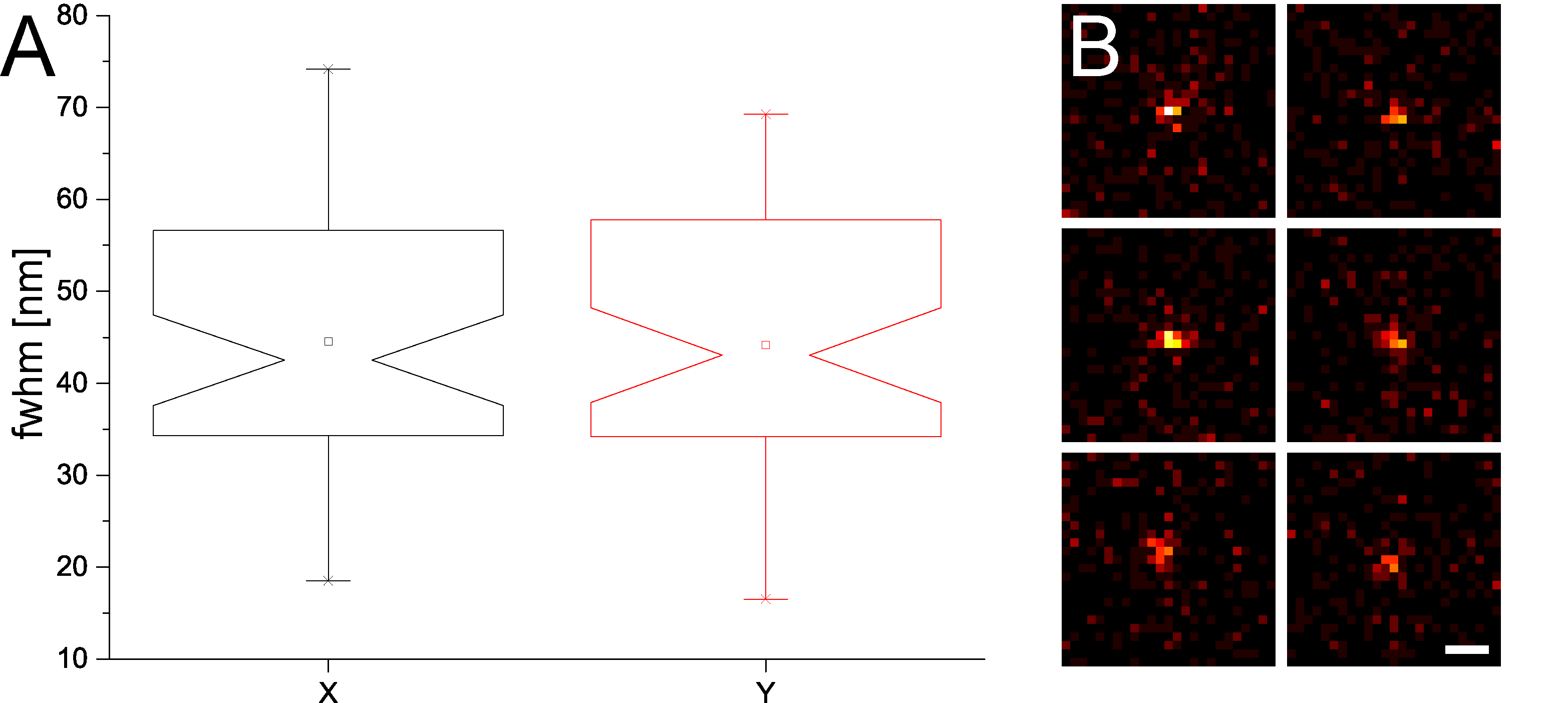

Supplement: Figure S1 — Measured diameter of background clusters taken from Figure 2 . (A) The full width at half maximum (fwhm) across the x and the y axes of more than 100 individual clusters was determined. Note that also background clusters that were physically larger than the resolution of the microscope were analyzed. Therefore the determined average fwhm may be worse than the actual resolution of the microscope used. (B) Representative background clusters. Scale bar: 100 nm. (TIF) [file pone.0101563.s001.tif]

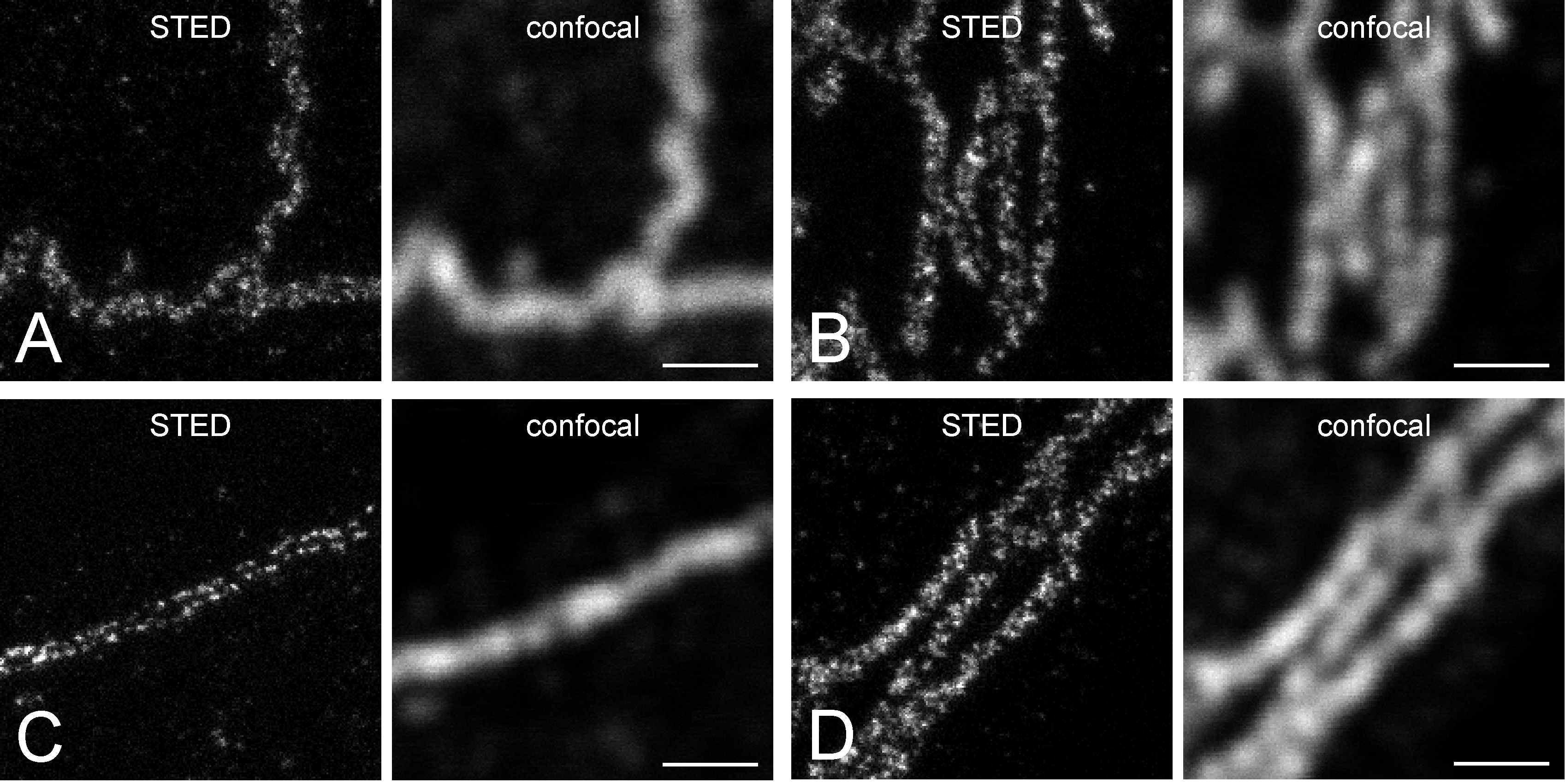

Supplement: Figure S2 — Sub-mitochondrial protein distributions in cultured human cells recorded with a STED (left) and confocal (right) microscope. Detail of mitochondria decorated with antisera against Tom20 (A), aconitase (B), Mic60/mitofilin (C), or cyclophilin D (D). Human primary fibroblasts (A,C) or U2OS (human bone osteosarcoma epithelial cells) (B,D). Scale bars: 500 nm. (TIF) [file pone.0101563.s002.tif]

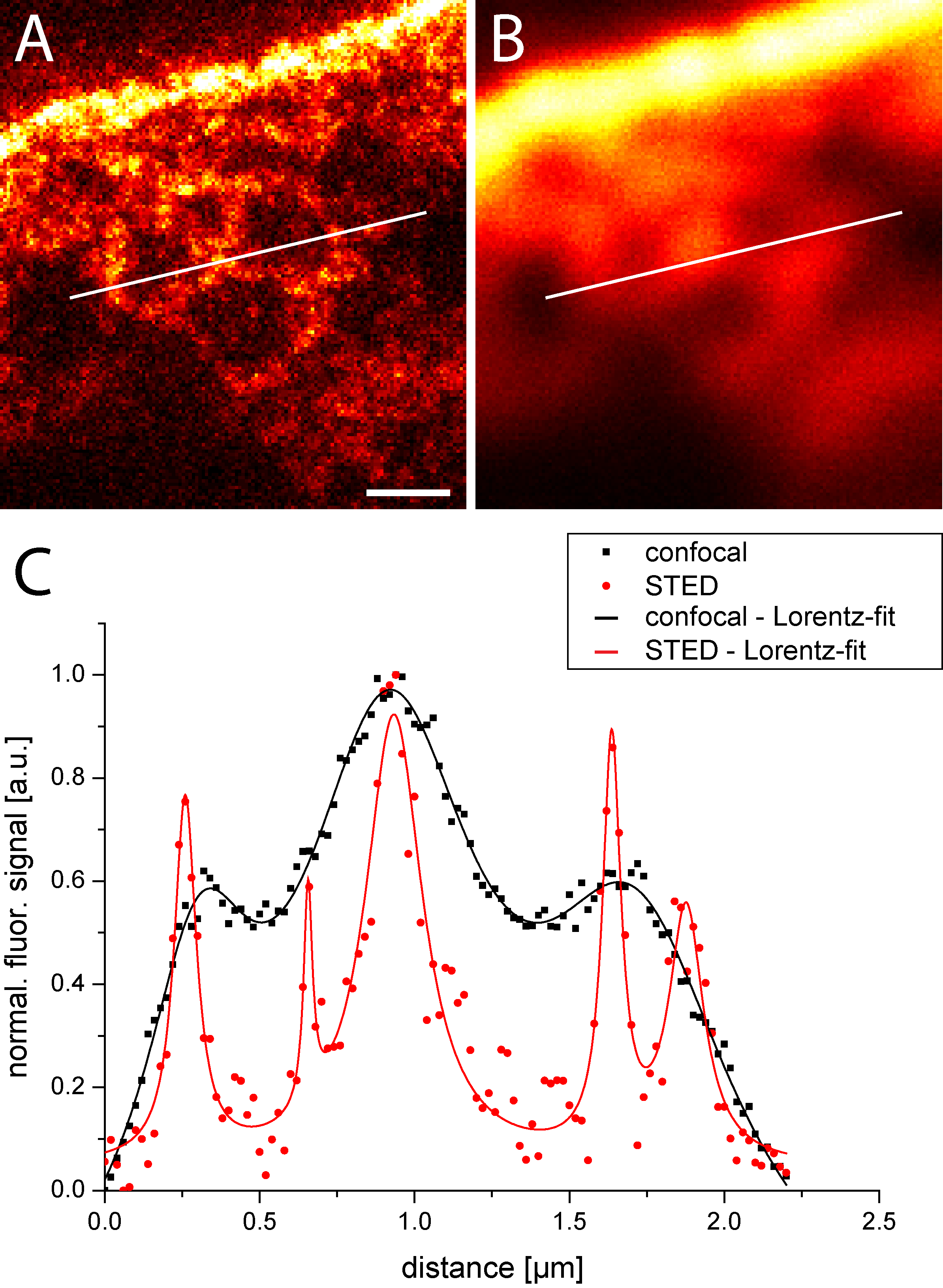

Supplement: Figure S3 — Line profile through a region taken from Figure 3F . The STED super-resolution image (A) reveals HER2 positive vesicle-like structures that are blurred in the corresponding confocal image (B). (C) Normalized fluorescence signal intensity profiles along the indicated lines in the STED (red) and the confocal (black) images. The scatter plot shows the fluorescence signals averaged over 3 adjacent intensity profiles. The solid lines represent the corresponding fits using a Lorentz function. Scale bar: 500 nm. (TIF) [file pone.0101563.s003.tif]
